# Mining Data From Plasma Cell Differentiation Identified Novel Genes for Engineering of a Yeast Antibody Factory

**DOI:** 10.3389/fbioe.2020.00255

**Published:** 2020-03-31

**Authors:** Essi V. Koskela, Alina Gonzalez Salcedo, Mari A. Piirainen, Heidi A. Iivonen, Heidi Salminen, Alexander D. Frey

**Affiliations:** Department of Bioproducts and Biosystems, Aalto University, Espoo, Finland

**Keywords:** transcriptomics, plasma cell differentiation, antibody, *Saccharomyces cerevisiae*, synthetic biology

## Abstract

*Saccharomyces cerevisiae* is a common platform for production of therapeutic proteins, but it is not intrinsically suited for the manufacturing of antibodies. Antibodies are naturally produced by plasma cells (PCs) and studies conducted on PC differentiation provide a comprehensive blueprint for the cellular transformations needed to create an antibody factory. In this study we mined transcriptomics data from PC differentiation to improve antibody secretion by *S. cerevisiae*. Through data exploration, we identified several new target genes. We tested the effects of 14 genetic modifications belonging to different cellular processes on protein production. Four of the tested genes resulted in improved antibody expression. The ER stress sensor *IRE1* increased the final titer by 1.8-fold and smaller effects were observed with *PSA1*, *GOT1*, and *HUT1* increasing antibody titers by 1. 6-, 1. 4-, and 1.4-fold. When testing combinations of these genes, the highest increases were observed when co-expressing *IRE1* with *PSA1*, or *IRE1* with *PSA1* and *HUT1*, resulting in 3.8- and 3.1-fold higher antibody titers. In contrast, strains expressing *IRE1* alone or in combination with the other genes produced similar or lower levels of recombinantly expressed endogenous yeast acid phosphatase compared to the controls. Using a genetic UPR responsive GFP reporter construct, we show that *IRE1* acts through constitutive activation of the unfolded protein response. Moreover, the positive effect of *IRE1* expression was transferable to other antibody molecules. We demonstrate how data exploration from an evolutionary distant, but highly specialized cell type can pinpoint new genetic targets and provide a novel concept for rationalized cell engineering.

## Introduction

Baker’s yeast *Saccharomyces cerevisiae* has been successfully converted into cell factories for various biotechnological products. One of the most promising markets for microbial products is pharmaceutical proteins for therapeutic applications. *S. cerevisiae* is already a major platform for production of biopharmaceuticals, including over 20 different proteins ranging from vaccines and insulin products to growth hormones (Walsh, 2014). However, the market growth among biopharmaceuticals is concentrated on antibody products, which are currently produced in mammalian cell cultures (Walsh, 2014; Sanchez-Garcia et al., 2016). As the demand for antibodies continues to increase, efforts to develop alternative platforms to mammalian cells for manufacturing of antibodies have been extending to microbial hosts.

*Saccharomyces cerevisiae* is not intrinsically suited for therapeutic glycoprotein production, because the yeasts’ native N-glycan structures are structurally different from the N-glycans present on therapeutic antibodies. Advances in glycoengineering of different yeasts in general and of baker’s yeast in particular have demonstrated that production of antibodies with the desired N-glycan structure is feasible (Li et al., 2006; Jacobs P.P. et al., 2009; Nasab et al., 2013; Laukens et al., 2015; Piirainen et al., 2016). Besides approaches modifying the N-glycosylation pathway *in vivo*, also enzymatic *in vitro* transglycosylation of yeast produced Fc-fragments and full-length antibody was demonstrated as a feasible option (Wei et al., 2008; Liu et al., 2018). However, major other cellular obstacles still need to be overcome, in particular the limited capacity of *S. cerevisiae* to produce and secrete recombinant proteins and foremost the structurally complex full-length antibodies. Despite the fact that during the last two decades numerous studies have pinpointed bottlenecks along the secretory pathway (Idiris et al., 2010), up to now, only few studies addressed the problem of enhancing expression of antibody molecules, in particular of full-length antibodies. Increasing the size of the ER, populating the ER with chaperones or preventing missorting to the vacuoles have been identified as bottlenecks for antibody and antibody fragment expression (Shusta et al., 1998; Hackel et al., 2006; Rakestraw et al., 2009; de Ruijter et al., 2016a, b). Furthermore, designing secretory leaders specifically for antibody expression proved to be a successful strategy (Rakestraw et al., 2009). Metabolomics studies indicated also potential metabolic misbalances in antibody expressing strains (de Ruijter et al., 2017).

Antibodies are effective agents of the humoral immunity that are naturally produced by plasma cells (PCs). It was noted some time ago, that PCs outlay the guide map for genetic engineers to improve antibody production platforms, notably mammalian cells (Dinnis and James, 2005). PCs develop from naïve B cells, which first undergo antibody diversification and affinity maturation (Victora and Nussenzweig, 2012). After the mature B cell clone with the most efficient antibody is selected, it develops into a plasmablast (PB) and begins the plasmacytic phase of differentiation. Although PBs secrete low amounts of antibodies, the major preparations for high efficiency antibody production occur during the plasmacytic phase (Shaffer et al., 2004; Oracki et al., 2010). PCs transform their cellular machinery completely to support antibody production, but not all of the metabolic and cellular changes needed might be obvious *per se*.

Nature has explicated the cellular design for effective secretion. We have shown previously that adapting selected features from PCs, such as their expanded ER or overexpression of mammalian chaperons successfully increased the antibody secretion capacity of *S. cerevisiae* cells (de Ruijter et al., 2016b; Koskela et al., 2017). Capitalizing on this natural expression system, we explored transcriptomics data sets from PC differentiation to identify novel candidates to be used as engineering targets to improve full-length antibody secretion in yeast. We selected differentially regulated genes and manipulated the expression levels of their corresponding yeast homologs. The four genes *IRE1*, *GOT1*, *HUT1*, and *PSA1* when expressed alone or in combinations were shown to be beneficial for antibody secretion in yeast. Further experiments established that the cell engineering strategy is applicable to the expression of other antibody molecules but not to the expression of the endogenous yeast acid phosphatase (AP).

## Materials and Methods

### Data Analysis Workflow for Plasma Cell Differentiation Phases

Data sets with GEO accession numbers GSE36975 (Le Gallou et al., 2012) and GSE41208 (Cocco et al., 2012) were downloaded to the R software environment by using the GEOquery-package (Davis and Meltzer, 2007). Both data sets were log2-transformed and GSE36975 was pre-processed with affyPLM-package (Bolstad, 2004) for quantile normalization. Probes without an EntrezID were removed from both data sets. R-package illuminaHumanv4.db (Dunning et al., 2015) was used for mapping probe annotations on the common microarray platform of the data sets. The conducted sample comparisons are depicted in the Supplementary Figure S1. In total four comparisons were made from PB phase (G1, G3, C2, and C1 in Supplementary Figure S1) and two from PC differentiation phase (C3 and C4 in Supplementary Figure S1). Differentially expressed genes were determined by using Student’s *t*-test and a *p*-value cutoff of 0.05. The differentially expressed genes were divided as follows: genes that had a mean fold-change >0 between the final and earlier states were considered to be upregulated and if mean fold-change was <0, the gene was considered as down-regulated. In the case of PB-specific comparisons, intersect of gene groups from the different data sets was selected in both subgroups (up- and downregulated).

The selected human EntrezIDs were converted to their *S. cerevisiae* homolog EntrezIDs when applicable with the idConverter()-function provided by AnnotationDbi (Pages et al., 2016) and enabled by organism-specific annotation packages org.Sc.sgd.db (Carlson, 2016) and hom.Hs.inp.db (Carlson and Pages, 2015). The total number of possible homologs from the microarray analysis was 1,364, covering approximately 25% of the yeast open reading frames. GO-term association analysis of the *S. cerevisiae* gene identifiers was realized with Cytoscape-software (Shannon et al., 2003) extended with the ClueGO-app (Bindea et al., 2009, 2013). Enrichment of the GO-terms was evaluated with a right-sided hypergeometric test without *p*-value correction from two clusters, the upregulated and downregulated subgroups.

### Cloning of Overexpression Constructs

All recombinant DNA work has been done using *Escherichia coli* TOP10 (Invitrogen) as the cloning host. All plasmids used in this work are summarized in Table 1. Plasmid pEK032 is based on the integrative plasmid pRS305K (Taxis and Knop, 2006) and contains the *S*. *cerevisiae PHO5* gene encoding yeast AP under control of *GAL1* promoter and *CYC1* terminator. The coding region of *PHO5* was amplified from genomic DNA with oligonucleotides OAF219 and OAF220 (Supplementary Table S1) and inserted into plasmid pRS425-GAL1 (Mumberg et al., 1994). The resulting *GAL1* promoter-*PHO5* gene-*CYC1* terminator fragment was inserted into *Sma*I *Bss*HII sites of pRS305K.

**TABLE 1:** List of plasmids used in this study.

Plasmids	Genotype	Reference/Source
pRS305K	Integrative plasmid, *LEU2* site, G418 selection marker	Taxis and Knop, 2006
pRS413-TEF	Low-copy number plasmid, *HIS3* selection marker, *TEF* promoter and *CYC1* terminator	Mumberg et al., 1995
pRS414	Low-copy number plasmid, *TRP1* selection marker	Mumberg et al., 1995
pRS415-TEF	Low-copy number plasmid, *LEU2* selection marker, *TEF* promoter and *CYC1* terminator	Mumberg et al., 1995
pRS416-GAL1	Low-copy number plasmid, *URA3* selection marker, *GAL1* promoter and *CYC1* terminator	Mumberg et al., 1994
pKK1	Low-copy number plasmid, *LEU2* selection marker, *PGK1* promoter and *PGK1* terminator	Valkonen et al., 2003a
pMS109	Derivative of pKK1, expression of intronless *HAC1*^*i*^	Valkonen et al., 2003a
pEK32	Derivative of pRS305K, with *GAL1* promoter-*PHO5*-*CYC1* terminator	This work
pAX512	Derivative of pRS416-GAL1, expression of scFv-Fc fusion construct derived from HyHel-10 scFv	de Ruijter et al., 2017
pAX529	Derivative of pRS416-GAL1, expression of scFv-Fc fusion construct derived from Herceptin antibody	This work
pAX535	Derivative of pRS416-GAL1, expression of full-length antibody derived from Herceptin antibody, heavy and light chain under control *GAL1* promoter and *CYC1* terminator	This work
pAX538	Derivative of pRS416-GAL1, expression of full-length antibody derived from HyHel-10 scFv, heavy and light chain under control *GAL1* promoter and *CYC1* terminator	de Ruijter et al., 2017
pIRE1	Derivative of pRS415-TEF, expression of *IRE1*	This work
pALG5	Derivative of pRS415-TEF, expression of *ALG5*	This work
pALG7	Derivative of pRS415-TEF, expression of *ALG7*	This work
pBXI1	Derivative of pRS415-TEF, expression of *BXI1*	This work
pDER1	Derivative of pRS415-TEF, expression of *DER1*	This work
pEK52	Derivative of pRS415-TEF, expression of *GOT1*	This work
pGSH2	Derivative of pRS415-TEF, expression of *GSH2*	This work
pHUT1	Derivative of pRS415-TEF, expression of *HUT1*	This work
pPSA1	Derivative of pRS415-TEF, expression of *PSA1*	This work
pSEC24	Derivative of pRS415-TEF, expression of *SEC241*	This work
pMP29	Derivative of pIRE1 with *PDI1* promoter instead of *TEF* promoter	This work
pMP30	Derivative of pIRE1 with *KAR2* promoter instead of *TEF* promoter	This work
pAF12	Derivative of pRS413-TEF, expression of *HUT1*	This work
pAF13	Derivative of pRS413-TEF, expression of *PSA*	This work
pEK54	Derivative of pRS416-TEF, expression of *GOT1*	This work
pAF14	Derivative of pRS416-TEF, expression of *HUT1*	This work
pAF15	Derivative of pRS416-TEF, expression of *PSA*	This work
pAF18	Derivative of pRS414, *TEF* promoter-*PSA1*-*CYC1* terminator	This work

Plasmids pAX512, pAX529, pAX535, and pAX538 are based on the low-copy number plasmid pRS416-GAL1 and contain *URA3* selection marker. All antibody coding sequences were fused to wildtype *MAT*α pre- and propeptide (*MAT*α prepro) and were codon optimized for expression in *S*. *cerevisiae* (Geneart, Germany). Coding sequences were expressed under control of *GAL1* promoter. Plasmids pAX512 and pAX538 encoding single-chain F_v_-F_c_ (scFv-Fc) fusion and full-length IgG derived from anti-hen egg lysozyme Fab molecule (HyHEL-10) (Smith-Gill et al., 1984) have been described earlier (de Ruijter et al., 2017).

Plasmid pAX535 encoding full-length Herceptin antibody has been generated in two steps. First, *MAT*α prepro-light chain (APZ76731.1) sequence-*PGK1*-terminator and *MAT*α prepro-heavy chain (APZ76730.1) sequences were inserted into *Sac*I *Acc*65I sites of plasmids pRS416-GAL1 (Mumberg et al., 1994) generating plasmid pAX519 and pAX521. Then, the expression cassette comprising *GAL1* promoter-*MAT*α prepro-heavy chain-*CYC1* was inserted into *Pdi*I *Nde*I sites of pAX519 generating plasmid pAX535.

Plasmid pAX529 encodes a scFv-Fc construct based on the Herceptin antibody. V_L_ linked via three repeats of the sequence GGGS to the V_H_ sequence were fused to the human CH_2_ and CH_3_ sequences using a previously described linker region (Powers et al., 2001) and appended to *MAT*α prepro. The codon-optimized sequence was inserted into *Spe*I *Xho*I sites of pRS416-GAL1.

The coding regions of *IRE1, SEC24, BXI1*, and *DER1* were amplified from genomic DNA with oligonucleotides as indicated (Supplementary Table S1) and cloned into low-copy number plasmid pRS415-TEF (Mumberg et al., 1995) by using restriction enzymes *Spe*I and *Xho*I. For *IRE1* expression two modified plasmids were constructed by replacing the original *TEF1* promoter with the *PDI1* promoter and *KAR2* promoter generating plasmids pMP29 and pMP30, respectively. The cloning of original plasmids is described earlier (de Ruijter et al., 2016b).

The coding regions of selected genes (*ALG5*, *ALG7*, *GOT1*, *GSH21*, *HUT1*, *PSA1*, and *QRI1*) were amplified from yeast genomic DNA by using oligonucleotides as indicated (Supplementary Table S1). The resulting products were subjected to another PCR reaction, where complementary overhangs to the plasmid were created with oligonucleotides 21 and 22. PCR-products were cloned between *Xba*I and *Xho*I sites of pRS415-TEF vector (Mumberg et al., 1995) by using exonuclease and ligation-independent cloning (Koskela and Frey, 2015).

For co-expression of multiple genes (*GOT1*, *HUT1*, and *PSA1*) vectors comprising *URA3*, *HIS3*, and *TRP1* selection markers were generated. In brief, *TEF* promoter-gene-*CYC1* terminator cassettes were inserted into *Sac*I *Dra*III sites of pRS414 generating plasmids with *TRP1* selection markers. For generation of vectors comprising *URA3* and *HIS3* selection markers, the genes were excised from existing plasmids and inserted into *Spe*I *Xho*I sites of plasmids pRS416-GAL1 and pRS413-GAL1, respectively.

The plasmid pMS109 for expression of the intronless active form of *HAC1* (*HAC1*^*i*^) and its control plasmid pKK1 are described elsewhere (Valkonen et al., 2003a).

### Generation of Yeast Strains

All strains used in this study are derived from YEK18, a previously engineered W303α based strain, comprising an expression cassette for the light and heavy chains of the anti-CD20 antibody fused to the *PHO5* signal peptide sequence. Both coding sequences are under control of the GAL1-promoter and the construct was integrated into the *his3*-11,15 site (de Ruijter et al., 2016b).

Plasmids and disruption cassettes were transformed into the yeast cells by using the lithium-acetate method (Gietz et al., 1992). Gene disruptions were selected using YPD-agar medium, supplemented with 200 μg/mL G418 (Sigma-Aldrich, Helsinki, Finland) or 100 μg/mL nourseothricin (Jena Biosciences, Jena, Germany) and verified with colony-PCR. Plasmid containing transformants were selected on synthetic drop-out (SD) agar (0.67% yeast nitrogen base without amino acids) supplemented with a dropout mix lacking leucine, uracil, histidine or tryptophan or the required combinations.

A YEK18 derivative lacking the two proteases *PRB1* and *PEP4* was generated in two steps. *LoxP*-kanMX-*loxP* disruption modules for *PRB1* and *PEP4* were amplified from pUG6 (Güldener et al., 1996) with PCR using oligonucleotide pairs EK005/EK006 and OJR38/OJR39, respectively (Supplementary Table S1). After the first round of transformation, a Δ*prb1* deletion strain was obtained and confirmed with colony PCR. The KanMX cassette was removed using Cre-lox recombinase system as described (de Ruijter et al., 2016b). In a second round, deletion of the *PEP4* gene was achieved generating the strain YEK66.

For generation of the UPR reporter strain containing an UPRE-GFP reporter, linearized plasmid pDEP17 (Pincus et al., 2010) was integrated into *trp1-1* locus of YEK18 generating strain YMH13.

For generation of the anti-CD20 antibody and AP co-expressing strain YMH14, plasmid pEK32 was linearized with *Dra*III and integrated into *leu*2-3,112 locus of YEK18.

*LoxP*-kanMX-*loxP* disruption modules for *ATE1*, *DPH2*, and *UTR4* were amplified from pUG6 (Güldener et al., 1996) with PCR using oligonucleotides 1-6 (Supplementary Table S1) and transformed into YEK18 generating strains YEK70, YEK71, and YEK72. All strains are listed in Table 2.

**TABLE 2:** List of yeast strains used in this study.

Strain	Genotype	Reference/Source
W303α	MATα, *leu2*-3,112 *trp1*-1 *can1*-100 *ura3*-1 *ade2*-1 *his3*-11,15	ATCC 208353
YEK18	MATα, *leu2*-3,112 *trp1*-1 *can1*-100 *ura3*-1 *ade2*-1 Δ*his3*:(anti-CD20 antibody under *GAL1*):NatMX	de Ruijter et al., 2016b
YEK61	MATα, *leu2*-3,112 *trp1*-1 *can1*-100 *ura3*-1 *ade2*-1 Δ*his3*:(anti-CD20 antibody under *GAL1*):NatMX Δ*prb1:loxP*	This work
YEK66	MATα, *leu2*-3,112 *trp1*-1 *can1*-100 *ura3*-1 *ade2*-1 Δ*his3*:(anti-CD20 antibody under *GAL1*):NatMX Δ*prb1:loxP*Δ*pep4:loxP-KanMX-loxP*	This work
YEK70	MATα, *leu2*-3,112 *trp1*-1 *can1*-100 *ura3*-1 *ade2*-1 Δ*his3*:(anti-CD20 antibody under *GAL1*):NatMX Δ*ate1:loxP-KanMX-loxP*	This work
YEK71	MATα, *leu2*-3,112 *trp1*-1 *can1*-100 *ura3*-1 *ade2*-1 Δ*his3*:(anti-CD20 antibody under *GAL1*):NatMX Δ*dph2:loxP-KanMX-loxP*	This work
YEK72	MATα, *leu2*-3,112 *trp1*-1 *can1*-100 *ura3*-1 *ade2*-1 Δ*his3*:(anti-CD20 antibody under *GAL1*):NatMX Δ*utr4:loxP-KanMX-loxP*	This work
YMH13	MATα, *leu2*-3,112 *TRP1*:UPRE-GFP *can1*-100 *ura3*-1 *ade2*-1 Δ*his3*:(anti-CD20 antibody under *GAL1*):NatMX	This work
YMH14	MATα, Δ*leu2*:*GAL1*-*PHO5*-KanMX *trp1*-1 *can1*-100 *ura3*-1 *ade2*-1 Δ*his3*:(anti-CD20 antibody under *GAL1*):NatMX	This work

### Cultivation Procedures

Strains were inoculated into 2 mL of synthetic drop-out (SD) medium (0.67% yeast nitrogen base without amino acids supplemented with a dropout mix lacking the appropriate amino acids and uracil, with 2% raffinose as the carbon source). The precultures were grown at 30°C, 220 rpm for 16 h. Cells were collected at 3,000 rcf for 5 min and resuspended in H_2_O to obtain a cell suspension of 5 OD_600_ per mL. Each well of a 24-deep well plate was filled with 3.84 mL of the expression medium (SD-medium lacking appropriate amino acids and uracil supplemented with 2% raffinose, 20 mM sodium phosphate buffer, pH 6.5 and 50 μg/mL BSA) and was inoculated with 160 μl of cell suspension to yield a starting cell concentration of 0.2 OD_600_ per mL. The 24-deep well plate was incubated at 30°C, 220 rpm for 6 h and induced with galactose to final concentration of 0.5%. After 16 h of cultivation, OD_600_ was measured, the culture medium was adjusted to 1× PBT (135 mM NaCl, 2.5 mM KCl, 10 mM Na_2_HPO_4_, 1.75 mM KH_2_PO_4_, 0.05% Tween-20) and the cultures were centrifuged at 3,000 rcf for 10 min and the cleared supernatant was stored at −20°C until analysis. Remaining medium was discarded and 24-deep well plates containing pelleted cells were stored at −20°C until analysis.

Initial experiments in 96-deep well plate format were done essentially as described above but with 1 mL of culture volume and expression for 24 h.

Samples for UPRE-GFP measurement were centrifuged at 5,000 rcf for 1 min and the cell pellets were resuspended in H_2_O to reach 5 OD_600_ per mL. 200 μl of cell suspension were transferred to 96-well plates and fluorescence was measured with an Eon Microplate Spectrophotometer (BioTek, Winooski, VT, United States) using appropriate filter set.

Cultivation of UPR reporter strains for continuous GFP measurements were conducted in 96-well microplates using an Eon Microplate Spectrophotometer (BioTek, Winooski, VT, United States). Precultures were diluted to 0.1 OD_600_ per mL and 100 μl of cell suspension were transferred to each well. The plate was incubated at 30°C, 220 rpm for 6 h and induced with galactose to final concentration of 0.5%. For repressing conditions 0.5% glucose were added. Growth (OD_600_) and GFP expression were monitored every 15 min for up to 40 h.

### Enzyme-Linked Immunosorbent Assay and Acid Phosphatase Assay

Handling of the cleared supernatants and IgG titer determination with an enzyme-linked immunosorbent assay (ELISA) have been described previously (de Ruijter and Frey, 2015).

Secreted AP activity was measured from cleared supernatants collected prior to adjusting medium to 1× PBT. AP activity was measured using an endpoint method. Ten microliters of culture supernatants were dispensed in triplicates into a 96-well-assay plates. Prior to the assay, the plates were prewarmed for 5 min at 30°C. The assay was started by addition of 100 μL of 20 mM para-nitrophenyl-phosphate (pNPP) (Sigma) in 100 mM sodium acetate buffer, pH 4.2. The reactions were quenched by addition of 200 μL 2M Na_2_CO_3_ after 20 min of incubation at 30°C.

### SDS-PAGE and Immunoblotting

Pellets for western blotting were prepared as described earlier. Briefly, plates were thawed on ice, cells were resuspended in 1 mL H_2_O containing 1 mM of PMSF. Ten OD_600_ units of cells were transferred to Eppendorf tubes and the cells were pelleted at 10,000 rcf for 5 min, +4°C. After this, cells were lysed and prepared for SDS-PAGE analysis as described (de Ruijter and Frey, 2015).

Equal amounts of cell extracts were resolved on SDS-polyacrylamide gels. Separated proteins were transferred onto nitrocellulose membranes at 25 mA and room temperature. Equal transfer of samples was confirmed with Ponceau staining of membranes. All incubation and washing steps were performed in 1% non-fat dry milk in Tris-buffered saline containing 0.1% Tween 20. After transfer, membranes were incubated with anti-human IgG (Fc specific)-peroxidase labeled antibody produced in goat (Sigma) for heavy chain detection, and anti-Human kappa light chains (bound and free)-peroxidase antibody produced in goat (Sigma) for light chain detection. Chemiluminescent detection was performed with Supersignal West Pico Plus detection reagent (Pierce, Rockford, IL, United States).

## Results

### Cues From B Cell Differentiation for Yeast Cell Engineering

Nature has developed the most sophisticated and efficient antibody expression factory –PCs. PC’s antibody expression and secretion capacity is estimated at 1,000 molecules per second per cell (Hendershot and Sitia, 2004). PCs derive from mature B cells through a complex differentiation process that has been characterized in several genome-wide studies at transcriptome and proteome level (Van Anken et al., 2003; Shaffer et al., 2004; Romijn et al., 2005; Cocco et al., 2012; Le Gallou et al., 2012). We hypothesized that the acquired data sets provide an underexplored repository of engineering targets and provide a guide map to improve antibody expression and secretion. We initiated our approach by conducting a bioinformatics analysis of PC development. We limited our analysis to data sets that included distinct phases of the differentiation process and thus selected two transcriptomics data sets (Cocco et al., 2012; Le Gallou et al., 2012). Although the majority of PCs are short-lived (Tarlinton et al., 2008), we deliberately selected a data set to explore the phenotype of long-lived PCs, as this subpopulation is truly adapted to the secretory burden (Cocco et al., 2012). We divided the differentiation process into two separate phases for the analysis: from mature B-cells to PB phenotype, and from PB to PC (Supplementary Figure S1). Although most of the cellular changes occur in the latter phase, we were interested to investigate whether some underlying changes are already initiated in the PB phase. We determined differentially expressed genes common to both data sets, and roughly divided them to up- and downregulated based on the mean fold-changes. From the differentially expressed genes in the PB phase, we found 45 and 94 yeast homologs corresponding to genes in the up- and downregulated groups, respectively, and in the PC phase we identified 255 and 219 yeast homologs, respectively, from the up- or downregulated genes. The lower numbers in PB phase might be due to strict data integration between the two data sets that we did not employ for the PC phase.

**FIGURE 1:**
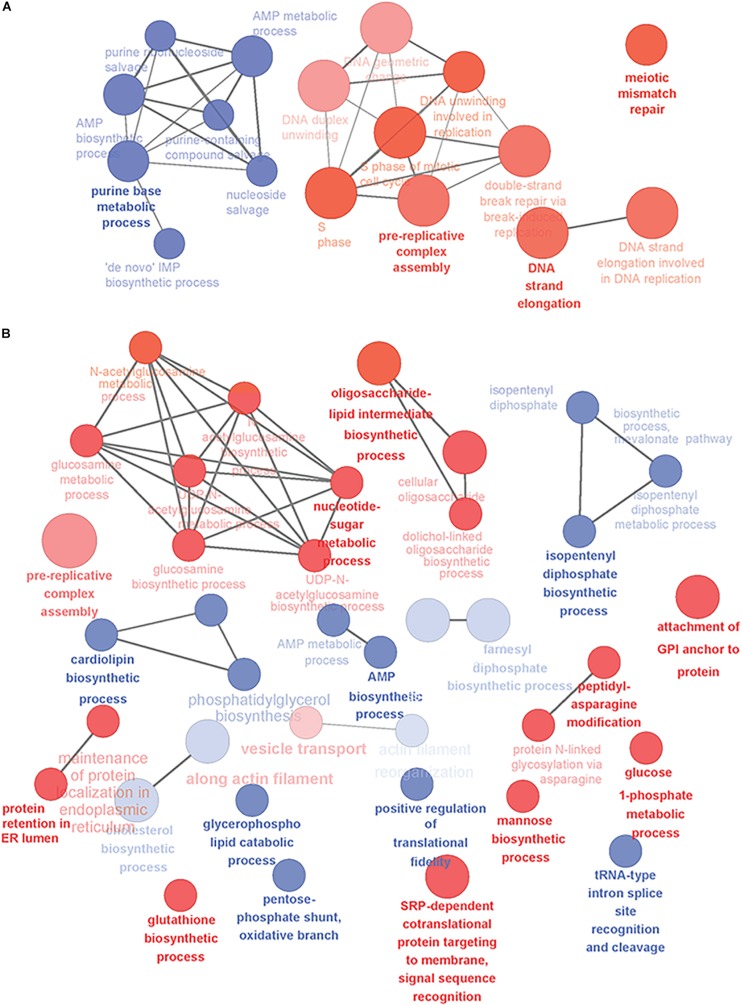
Overview of the yeast-relevant GO-terms displaying enrichment in plasma cell differentiation. Some of the enriched GO-terms of biological processes are visualized in **(A)** for early phases of plasma cell differentiation until the PB phenotype and **(B)** for plasmacytic phase of differentiation from PBs into long-lived plasma cells. Up-regulated processes are colored red and down-regulated blue. Size of the node reflects the number of genes identified for that process and the color intensity reflects the statistical significance of the enrichment. The showed networks are mainly descriptive and not directly comparable, as they were created with different settings of GO-levels, annotation requirements and network connectivity. Figure was created with Cytoscape and the ClueGO-app.

Next, we analyzed the yeast gene candidates with GO-term enrichment and some of the detected biological processes are illustrated in Figure 1. When mature B cells undergo selection and affinity maturation, the cell expressing the needed antibody will start to proliferate (Victora and Nussenzweig, 2012). This largely explains the domination of DNA replication and cell division related processes in the PB phase (Figure 1A). In contrast, the PC phase displayed differential regulation in several antibody expression and secretion related cellular processes illustrating the transformation needed for efficient protein secretion (Figure 1B). In general, upregulation of genes involved in translocation, lipid-linked oligosaccharide biosynthesis and protein N-glycosylation, nucleotide sugar metabolism and vesicle trafficking was prominent (Supplementary Table S2).

### Selecting Target Genes for Cell Engineering

To select the gene targets for experimental testing, we explored a detailed list of the enriched biological processes (Supplementary Table S2). In the quest to find potential engineering targets, we went through the gene candidates based on their GO-association. Keeping the risk of affecting viability in mind, we excluded genes associated with central biological processes, such as cell organization and division, basic metabolism, DNA, RNA, and cytoskeleton related terms. We excluded genes that have been reported to decrease fitness considerably (based on *Saccharomyces* genome database). In contrast to PC-phase, PB-phase comprised almost exclusively of essential processes. However, we decided to test the removal of *DPH2*, which participates in modifying translation initiation factors. All other candidates were derived from PC-phase.

In total, we selected 14 genetic modifications, where the type of modification, specifically deletion or overexpression, was largely determined by the direction of differential expression of the human homologs in the original analysis (Table 3). The selection of target genes to be overexpressed was biased toward genes with a direct or indirect link to process taking place along the secretory pathway, including genes involved in protein N-glycosylation (*ALG5*, *ALG7*, *PSA1*, and *QRI1*), transport (*SEC24* and *GOT1*), ER associated protein degradation and ER homeostasis (*BXI1*, *DER1*, *GSH2*, *HUT1*, and *IRE1*). The genes *IRE1*, *SEC24*, and *BXI1* had been identified in a preliminary version of the data analysis workflow of the same data sets. As targets for deletion we selected three genes, *ATE1*, *DPH2*, and *UTR4*, the function of these three genes has so far not been connected to recombinant protein production.

**TABLE 3:** Selected candidate genes and their functions.

Gene name	Modification	Function
*ALG5*	OE	UDP-glucose:dolichyl-phosphate glucosyltransferase; involved in asparagine-linked glycosylation in the endoplasmic reticulum
*ALG7*	OE	UDP-N-acetyl-glucosamine-1-P transferase; transfers GlcNAc-P from UDP-GlcNAc to Dol-P in the ER in the first step of the dolichol pathway of protein asparagine-linked glycosylation
*BXI1*	OE	Protein involved in apoptosis, localizes to ER and vacuole; may link the unfolded protein response to apoptosis
*DER1*	OE	ER membrane protein that promotes export of misfolded polypeptides, required for ER-associated protein degradation of misfolded or unassembled proteins
*GOT1*	OE	Homodimeric protein that is packaged into COPII vesicles; involved in vesicle trafficking between the ER and Golgi
*GSH2*	OE	Glutathione synthetase
*HUT1*	OE	Function unclear, has similarity to human UDP-galactose transporter UGTrel1
*IRE1*	OE	ER-resident transmembrane protein that initiates the unfolded protein response
*PSA1*	OE	GDP-mannose pyrophosphorylase (mannose-1-phosphate guanylyltransferase); synthesizes GDP-mannose from GTP and mannose-1-phosphate
*QRI1*	OE	UDP-N-acetylglucosamine pyrophosphorylase; catalyzes the formation of UDP-N-acetylglucosamine (UDP-GlcNAc)
*SEC24*	OE	Component of the COPII vesicle coat; required for cargo selection during vesicle formation in ER to Golgi transport
*ATE1*	KO	Arginyl-tRNA-protein transferase; catalyzes post-translational conjugation of arginine to the amino termini of acceptor proteins which are then subject to degradation via the N-end rule pathway
*DPH2*	KO	Protein required for synthesis of diphthamide
*UTR4*	KO	Involved in methionine salvage

*Target modifications were chosen to be either deletions (KO) or overexpressions (OE) largely based on the direction of the change in the plasma cell data sets. Functions of the genes were obtained from the Saccharomyces genome database (http://www.yeastgenome.org/).*

### Initial Screening of the Candidate Genes

**FIGURE 2:**
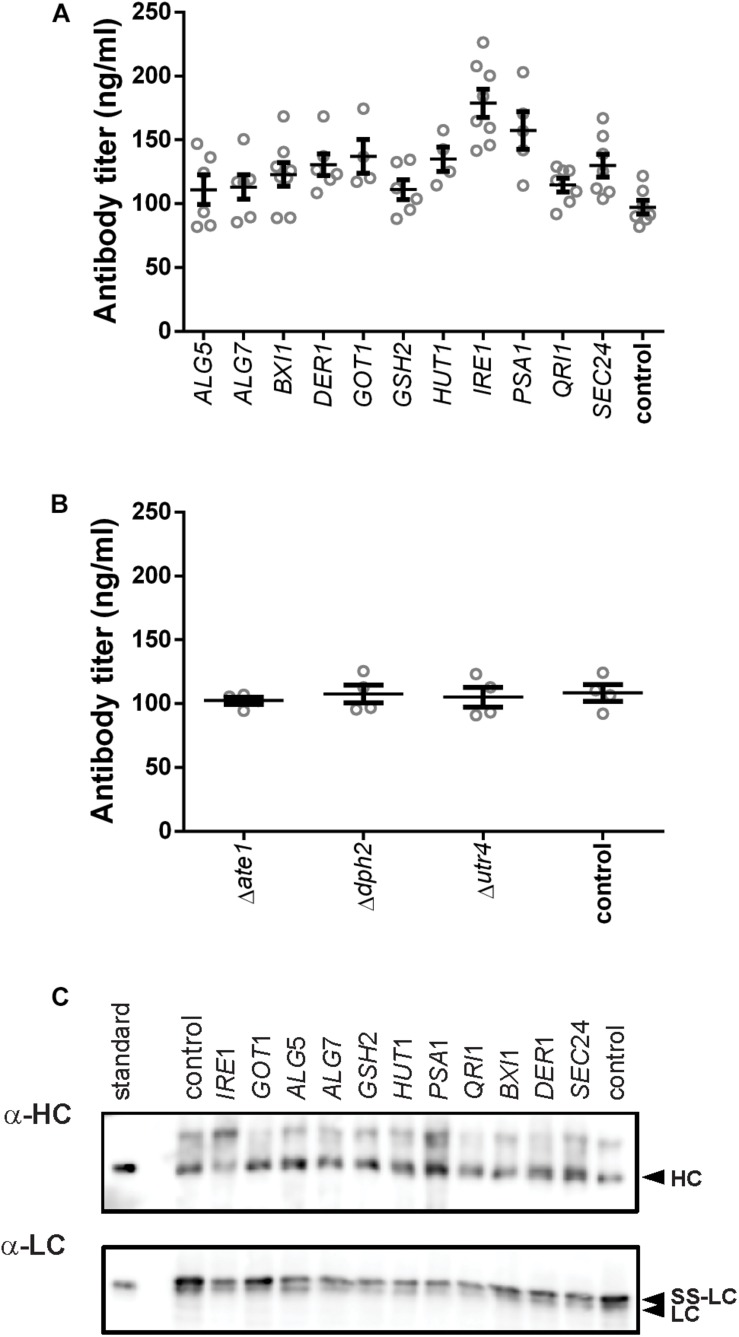
Effects of the tested genes on IgG secretion and expression. Mean antibody titers are displayed for each genetic modification for overexpression **(A)** and deletion strains **(B)**. Overexpressed or deleted genes are indicated below the charts. Overexpression and corresponding control strains were derived from YEK66. Strains were grown in SD medium lacking leucine. Deletion strains are derived from YEK18 and were grown in complete SD medium. Data points of biological replicates together with mean and standard error are shown. *N* = 4–8. **(C)** Immunoblot analysis of cell extracts after 16 h of expression.

After defining the modifications, we analyzed the effects of their expression on antibody secretion in an IgG-expressing yeast strain, which had the expression cassette for an anti-CD20 antibody under the control of the *GAL1*-promoter integrated into its genome. For overexpression of genes, we amplified the coding region from genomic DNA and cloned them into an episomal vector under control of the *TEF*-promoter. Knock-out strains were created by deleting the complete open reading frames from the genome. After initial experiments in 96-deep well plates, all experiments were conducted in 24-deep well plate format. Antibody titers in culture supernatants were measured 16 h after induction. Four out of the eleven overexpressed genes had a positive effect on antibody secretion. The highest effect was obtained by expression of the UPR sensor *IRE1* increasing the final titer by 1.8-fold reaching 178.79 ± 10.88 μg/L, smaller positive effects were observed with *PSA1*, *GOT1*, and *HUT1* increasing antibody titers by 1. 6-, 1. 4-, and 1.4-fold, respectively, and reaching 157.40 ± 14.75 μg/L, 137.10 ± 13.18 μg/L, and 134.90 ± 9.50 μg/L, respectively (Figure 2A and Supplementary Table S3). In order to differentiate between an improved expression of the IgG and improved secretion, we compared the intracellular levels of heavy and light chains using immunoblotting. The data indicated that although variations in the amounts of intracellular heavy chains are visible, there is no indication that substantial amounts of IgG accumulated within the cells (Figure 2C).

Although deletion of *DPH2* improved titers in the initial 96-well screening format (Supplementary Figure S2), antibody secretion in the 24-well format did not result in any difference compared to the control strain (Figure 2B). None of the other two deletions resulted in any changes in the antibody expression or growth. Therefore, the three deletions strains were excluded from further experiments.

### Synergistic Effects of Gene Overexpression on Antibody Secretion

**FIGURE 3:**
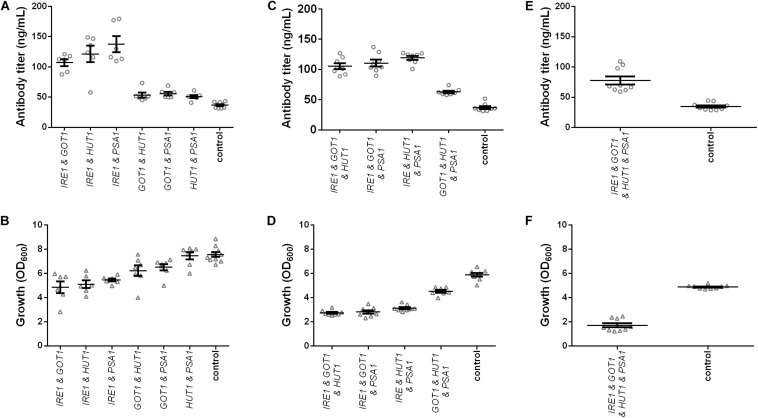
Effects of combined expression of *IRE1*, *GOT1*, *HUT1*, and *PSA1* genes on IgG secretion and growth. Mean antibody titers and final cell density are displayed for each genetic modification for strains expressing combinations of two **(A,B)**, three **(C,D)**, and four genes **(E,F)**. Overexpressed genes are indicated below the charts. Overexpression and corresponding control strains were derived from YEK66 and contain two, three, or four low copy number plasmids. Control strains contain corresponding number of empty plasmids. Strains were grown in SD medium lacking the appropriate amino acids (leucine, histidine, and tryptophan) and uracil. Data points of biological replicates together with mean and standard error are shown. *N* = 6–9.

Next, we created YEK66 strains for pairwise expression of *IRE1*, *GOT1*, *HUT1*, and *PSA1* and tested their effects on antibody expression. Of the six strains, the strains co-expressing *IRE1* together with either *GOT1*, *HUT1*, or *PSA1* strongly increased antibody titers but also resulted in reduced final cell densities (Figures 3A,B and Supplementary Table S3). The highest titers were obtained in the strain co-expressing *IRE1* and *PSA1* reaching a mean titer of 137.68 ± 13.19 μg/L being 3.77-fold higher than the corresponding control (36.56 ± 1.63 μg/L). The two other pairs including *IRE1* expression construct, reached final titers of 121.3 ± 13.80 μg/L (*IRE1* and *HUT1*) and 107.08 ± 5.72 μg/L (*IRE1* and *GOT1*) being 3.2- and 2.9-fold higher than the corresponding control strain. The other tested pairs resulted in smaller increases in final antibody titers ranging between 1.3 and 1.5-fold, but also in less than 20% reduction in final OD_600_ compared to the control (Figures 3A,B and Supplementary Table S3).

In order to further improve antibody titers, co-expression of three and four genes at a time was tested. Despite the negative effects on growth, co-expression of three genes led to approximately threefold higher final antibody titers for strains co-expressing *IRE1* together with either *GOT1* and *HUT1*, *GOT1* and *PSA1*, or *HUT1* and *PSA1* reaching final titers of 105.10 ± 4.80 μg/L, 110.50 ± 5.56 μg/L, 119.20 ± 3.19 μg/L (Figure 3C and Supplementary Table S3). However, also co-expression of *GOT1*, *HUT1*, and *PSA1* resulted in a 1.6-fold increase in antibody concentration reaching 62.68 ± 2.13 μg/L. In the final experiments, all four genes were co-expressed. Antibody titer was 2.2-fold higher compared to the control strain and reached 77.61 ± 6.65 μg/L. However, growth was severely reduced reaching only 35% of the cell density of the corresponding control (Figures 3E,F and Supplementary Table S3).

On a per cell base, antibody productivity increased 6.5-fold for the strains co-expressing all four genes, 6.4-fold for the strain co-expressing *IRE1*, *GOT1*, and *PSA1* and 5.8-fold for the strain co-expressing *IRE1* and *HUT1* compared to the corresponding control strains (Supplementary Figure S3).

**FIGURE 4:**
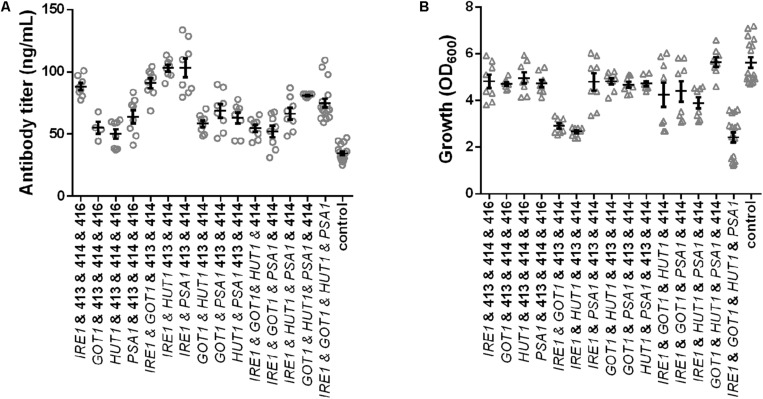
Effects of the selected genes on IgG secretion and growth. Mean antibody titers **(A)** and final cell density **(B)** are displayed for each strain expressing one or selected combinations of two, three, and four genes. Overexpressed genes are indicated below the charts. Overexpression and corresponding control strains were derived from YEK66 and all strains contain four low copy number plasmids. Empty plasmids are derived from pRS plasmid series and are labeled 413, 414, 415, and 416. Strains were grown in SD medium lacking leucine, histidine, tryptophan, and uracil. Data points of biological replicates together with mean and standard error are shown. *N* = 8–12.

In order to eliminate the variation in plasmid construct number and assess the contributions of each gene on production and growth a final set of strains was generated, which all contained four plasmids. Expression strains were complemented with the missing empty plasmids. Overall, we generated four strains expressing single genes, six strains expressing pairs, four strains expressing all possible combinations of three genes and one strain expressing all four genes and a control strain harboring four empty plasmids. All strains were grown under identical conditions and final cell density and antibody titers were determined. The measured antibody concentrations followed a similar pattern as observed in the earlier results, although the absolute titers were reduced (Figure 4A and Supplementary Table S3). The three best strains expressed *IRE1* alone, *IRE1* and *HUT1* and *IRE1* and *PSA1* and reached antibody titers of 88.03 ± 2.85 μg/L, 103.00 ± 2.63 μg/L, 103.10 ± 7.55 μg/L, respectively, compared to the control strain. As expected, the presence of the four plasmids lowered final OD_600_. Expression of single genes had only minor negative effects on growth compared to the control. With the exception of one strain (*IRE1* and *PSA1*), expression of *IRE1* led to a noticeable growth phenotype. In contrast, for strains expressing gene combinations not including *IRE1*, the negative effects on growth were smaller (Figure 4B and Supplementary Table S3).

Therefore, in order to test whether the expression of *IRE1* can be optimized two additional constructs were generated where the *TEF* promoter was replaced with the *KAR2* and *PDI1* promoter, respectively. We previously showed that these promoters are less strong and were suitable for expression of molecular chaperones (de Ruijter et al., 2016b). When *IRE1* was expressed alone under control of the three different promoters, the antibody titers and growth were very similar (Supplementary Figure S4). When combined with *GOT1*, *HUT1*, and *PSA1* expression plasmids, antibody titers were similar in all three strains expressing *IRE1*, irrespective of the promoter controlling expression (Supplementary Figure S4). However, growth of the three strains varied, obtaining the lowest final OD_600_ with expression of *IRE1* driven by *PDI1* promoter, but none of the constructs alleviated the growth phenotype obtained with the initial construct.

### Transferability of Effects to Other Antibodies

**FIGURE 5:**
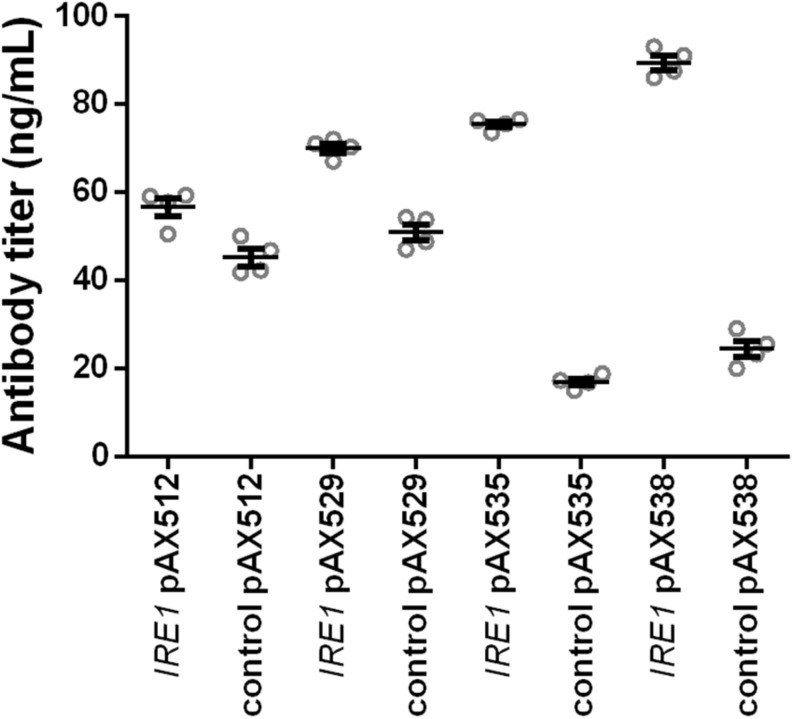
Expression of *IRE1* increases secretion of various IgGs. Two full-length and two scFv-Fc antibodies were co-expressed with *IRE1* or a control plasmid. Mean antibody titers are displayed for each strain expressing scFv-Fc antibodies (pAX512 and pAX529) or full-length antibodies (pAX535 and pAX538). Overexpression and corresponding control strains were derived from W303α and all strains contain two low copy number plasmids. Antibody expression is under control of P_*GAL*1_. Strains were grown in SD medium lacking leucine and uracil. Data points of biological replicates together with mean and standard error are shown. *N* = 4.

In order to test whether the positive effects were restricted to the anti-CD20 antibody expressed in the screening strains, we evaluated whether the positive effects are more general. Therefore, we included four additional antibody constructs, two full-length antibodies (anti-hen egg lysozyme and Herceptin) and two single-chain-Fc constructs. In contrast to the anti-CD20 antibody, Matα signal sequence is utilized to direct these molecules to the secretory pathway. As *IRE1* expression was identified as the main contributor to the positive effects, we limited the experiments to co-expressing *IRE1* together with the antibody constructs in the wild-type strain W303α applying our standard 24-well plate cultivation protocol. The control strains contained the corresponding empty plasmid. The expression of all four antibodies was improved by co-expression of *IRE1*, although the magnitude of the effect varied. The relative improvement was higher for the full-length antibodies than for the less complex scFv-Fc’s (Figure 5 and Supplementary Table S3). *IRE1* expression led to 1.3- and 1.4-fold higher expression of scFv-Fc’s and to 4.5- and 3.7-fold higher expression of full-length Herceptin antibody and full-length HyHEL antibody reaching a final titers of 75.44 ± 0.68 μg/L and 89.38 ± 1.60 μg/L, respectively, confirming that the effects are transferable to other antibody molecules.

### Expression of the Endogenous Protein Acid Phosphatase Is Not Improved by the Cell Engineering Approach

Next we wondered whether the positive effects are specific for recombinant antibodies or also observable for the expression of other proteins. In order to test this we generated a yeast strain harboring the expression cassette for the anti-CD20 antibody under control of *GAL1* promoter in the *HIS3* locus and an additional expression cassette for the endogenous AP expressing *PHO5* gene under control of *GAL1* promoter in the *LEU2* locus. Preliminary tests with this strain showed that the AP activity increased with increasing galactose concentration from 0 to 4%. In contrast, IgG secretion was limited and the titers reached a plateau above an induction level of 1% galactose (Supplementary Figure S5). Thus, while secretion capacity of antibodies is clearly limited, the secretion of the endogenous AP activity is not limited under the tested conditions.

**FIGURE 6:**
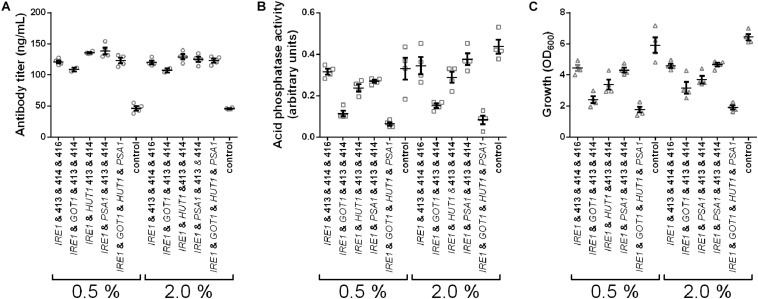
*IRE1*, *GOT1*, *HUT1*, and *PSA1* enhance secretion of IgG but not of overexpressed endogenous AP. Antibody titers **(A)**, secreted AP activity **(B)**, and final cell density **(C)** are displayed for each strain expressing one or selected combinations of two, or all four genes. Overexpressed genes are indicated below the charts. All strains were derived from YMH14 and contain four low copy number plasmids. Expression of antibody and AP are under control of P_*GAL*1_. Expression strains were grown in SD medium lacking leucine, histidine, tryptophan, and uracil. Protein expression was induced with 0.5 and 2.0% galactose. Data points of biological replicates together with mean and standard error are shown. *N* = 4.

In the experiments we tested expression of *IRE1*, co-expression of *IRE1* with *GOT1*, *HUT1*, or *PSA1* and co-expression of all four genes and measured secreted antibody titers and AP activity. Antibody secretion and growth were influenced in similar ways as observed earlier, yielding increased titers, but lower final OD_600_ for the engineered strains (Figure 6). Furthermore, higher induction levels did not increase antibody titers. In contrast to antibody concentrations, the secreted AP activity was highest in the control strain and reduced to different levels in the other strains. Moreover, AP activity increased when using the higher induction levels. Notably, the measured AP activities correlated well with the final OD_600_ (Figures 6B,C). Overall, the results imply that the cell engineering approach is highly specific for increasing antibody expression.

### Expression of *IRE1* Activates the Unfolded Protein Response

**FIGURE 7:**
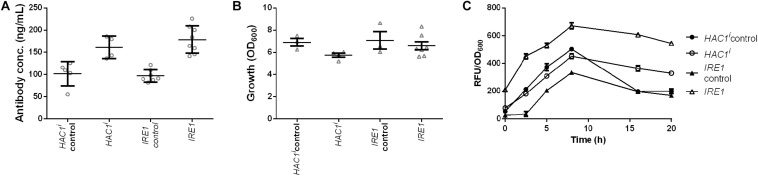
Expression of UPR sensor and UPR specific transcription factor Hac1p enhances antibody production. Mean antibody titers **(A)**, final cell density **(B)**, and UPR activation **(C)** are displayed for strains constitutively expressing active spliced *HAC1*^*i*^ and *IRE1*. For each expression construct the corresponding control plasmid was included. All strains were derived from YEK66. Strains were grown in SD medium lacking leucine. Data points of biological replicates together with mean and standard error are shown. *N* = 4–8.

Induction of premature or constant UPR has been shown to enhance secretion, but the induction was established with overexpression of *HAC1*^*i*^ encoding active spliced Hac1p, the protein downstream of Ire1p (Sidrauski and Walter, 1997; Valkonen et al., 2003a). We set out to test whether our novel gene identified through the data exploration would have similar effects on IgG secretion as this known approach. Two strains together with their corresponding control strains were generated and effects of Hac1p and Ire1p on antibody secretion and growth were analyzed. Our data showed that expression of both the sensor kinase as well as the active transcription factor increased antibody secretion with only a small effect on growth (Figure 7 and Supplementary Table S3). Antibody concentrations increased 1.6- and 1.8-fold, whereas final OD_600_ reached 83 and 93% of the corresponding control strains for *HAC1*^*i*^ and *IRE1* expression strains, respectively (Figures 7A,B). Next we investigated UPR induction in the antibody expressing strain using a previously published UPR responsive GFP reporter construct (UPRE-GFP) (Pincus et al., 2010). *HAC1*^*i*^ expression leads to constitutive UPR induction and thus served as a reference. We compared the *HAC1*^*i*^ and *IRE1* expression strains in two different settings, in repressive glucose medium inhibiting antibody expression and under inducing conditions in microtiter plate cultivations. As expected *HAC1* expression led to activation of UPR under both repressive and inducing conditions. A similar response was observed for *IRE1* expressing strain, indicating that overexpression of UPR sensor leads to activation of the stress response irrespective of the antibody expression. The control strains expressing antibody alone showed detectable responses only when grown on galactose (Supplementary Figure S6). Next we compared UPR induction in our standard expression set-up. Based on the preliminary data, we selected five time points for measuring UPR activation. Samples were measured at time points 0, 2.5, 5, 8, and 16 h after induction. Overexpression of UPR signaling proteins induced measurable UPR. UPR induction was higher in both strains expressing the UPR sensing and signaling molecules (Figure 7).

## Discussion

In this study we demonstrated the potential of using PC differentiation as a blueprint to guide cell engineering. Through data exploration, we identified 300 and 315 yeast homologs in the group of up- and downregulated genes, respectively. From these genes, we selected 14 for experimental testing. Out of the 14 target genes, the manipulation of four genes resulted in positive effects on antibody expression. All four genes were selected from the group of upregulated genes during PC differentiation and were therefore overexpressed in our experiments.

Especially the analysis of the PC-phase unearthed several genes that have been successfully tested in engineering recombinant protein production. For example, the ER chaperone BiP (yeast *KAR2*) and the nucleotide exchange factor SIL1 (yeast *SIL1*) were in the upregulated group associated to several of the identified biological processes (Supplementary Table S2). Both functionalities, human BiP or *KAR2* and yeast *SIL1* have been overexpressed in antibody producing *S. cerevisiae* strains (Shusta et al., 1998; Hackel et al., 2006; de Ruijter et al., 2016b). Moreover, the homologs of the vacuolar sorting proteins Vps41p and Vps45p appeared in the category of downregulated genes, corresponding to previous reports where deletion of several of the *VPS* genes has been found beneficial for recombinant protein secretion (Zhang et al., 2001; Rakestraw et al., 2009; de Ruijter et al., 2016a).

The strongest effect was obtained by expression of the UPR sensor, the transmembrane protein Ire1p. The positive effect of *IRE1* expression was confirmed by co-expression with four different antibody molecules showing that its positive effect was not restricted to enhancing secretion of the anti-CD20 antibody in the screening strain but was applicable to other antibody molecules and formats (Figure 5). The effects were notably higher with the structurally more complex tetrameric full-length IgGs than the dimeric scFv-Fc constructs. Moreover, the effects of *IRE1* on antibody secretion was independent of the signal sequence used, the 19 amino acids long *PHO5* derived signal peptide in the case of anti-CD20 antibody and the Matα signal sequence for the other four antibody molecules. It is interesting to note in this context that expression of different antibody formats led to distinctive reshaping of intracellular metabolome (de Ruijter et al., 2017). Furthermore, relative rate of secretion decreased with increasing complexity of the protein in *Pichia pastoris* (Love et al., 2012). Furthermore, it was reported that *IRE1* expression led to a higher intracellular level of a Hepatitis B small antigen GFP fusion in yeast (Sheng et al., 2017). In contrast, co-expression of the endogenous AP did not benefit from *IRE1* expression under the tested conditions (Figure 6). Ire1p senses and signals ER stress by splicing the *HAC1* transcript into its active form. Activation of Ire1p is a two-stage process in which first Kar2p is released followed by binding of unfolded proteins inducing oligomerization of Ire1p (Kimata et al., 2007). Experiments using a UPRE-GFP reporter showed that *IRE1* expression led to a higher basal GFP level under repressing conditions, indicating that UPR is preactivated. It is plausible that the overexpression of *IRE1* shifts the ratio between Kar2p and Ire1p, therefore making Ire1p more susceptible to ER stress. Thus, our finding is in contrast to earlier results, where the overexpression of *IRE1* from a high-copy number plasmid did not result in UPR activation as shown through analyzing *HAC1* splicing (Kimata et al., 2007).

Genetic induction of UPR by overexpression of the spliced transcript of *HAC1*, *HAC1*^*i*^, has been shown to enhance protein secretion in yeasts and fungi (Valkonen et al., 2003a, b; Gasser et al., 2007; Payne et al., 2008). Therefore, we have also compared these two approaches to induce UPR. Our data indicate that expression of *IRE1* is slightly more successful in increasing antibody secretion relative to *HAC1*^*i*^ expression. In contrast to expression of active Hac1p, cells overexpressing Ire1p can putatively still sense physiological ER stress, which can be important in order to tune the UPR according to cellular needs. Although replicating the events taking place in PCs, induction of UPR via expression of either *IRE1* or *HAC1*^*i*^ resembles more a shotgun approach than a designed genetic modification.

The essential protein Psa1p catalyzes the last step of the GDP-mannose biosynthesis. GDP-mannose is used as substrate in the cytoplasmic steps of the LLO biosynthesis, in all mannosylation steps in the Golgi apparatus and serves as a donor for dolichol-phosphate mannose synthesis. Expression of the *PSA1* gene in *S*. *cerevisiae* was shown earlier to complement defects in the LLO biosynthesis rescuing temperature sensitive mutations of *alg1*, *alg2*, and *dpm1* genes and restoring protein N-glycosylation (Janik et al., 2003). As the expressed antibody is not a heavily glycosylated protein, having only one glycosylation site in each heavy chain, it is likely that observed improvements are due to indirect effects on cellular glycosylation status and viability. Interestingly, expression of *Kluyveromyces lactis* homolog KlPsa1 enhanced secretion of the non-glycosylated protein human serum albumin and of the N-glycosylated *Arxula adeninivorans* glucoamylase in *K. lactis* (Uccelletti et al., 2005). Opposite effects on protein secretion were reported when studying a GDP-mannose phosphorylase in *Hansenula polymorpha* where inactivation of the gene led to an increased secretion of two proteins (Agaphonov et al., 2001). Taking together the previous and our current results, the molecular bases underlying the observed increased protein secretion are not yet clear. Moreover, the other genes with links to protein glycosylation, *ALG5*, *ALG7*, and *QRI1* did not result in any effects.

*GOT1* overexpression led to a moderate increase in antibody expression. The function of Got1p in the COPII-coated vesicles is less defined (Conchon et al., 1999; Malsam et al., 2008; Lorente-Rodríguez et al., 2009). Conchon et al. (1999) suggested that Got1p contributes to vesicle fusion on the *cis*-Golgi membrane, but Lorente-Rodríguez et al. (2009) showed that most of Got1p localizes to the ER. Strong overexpression of Got1p induced elaboration of ER membranes, which Lorente-Rodríguez et al. (2009) inferred to be caused by a block in secretion. We show here that overexpression of Got1p in fact increased secretion, which implies that Got1p is an active player in vesicle trafficking.

The cellular function of the multi-pass membrane protein Hut1p is the least known of the four proteins. Due to sequence similarity with the human UDP-galactose transporter it had been studied in the context of cellular glycosylation (Kainuma et al., 2001), but other studies on Hut1p from *S*. *cerevisiae* and *Schizosaccharomyces cerevisiae* implied that Hut1p is involved in protein folding in the ER maintaining optimal conditions for folding of secretory pathway proteins (Nakanishi et al., 2001). This hypothesis was corroborated by genetic interaction of *HUT1* with *ERO1* and increased sensitivity to DTT of a Δ*hut1* deletion strain.

When combining the expression of multiple genes, the highest increases were observed combining *IRE1* with *PSA1* or *IRE1* with *PSA1* and *HUT1*, reaching 3.8- and 3.1-fold higher antibody titers compared to the corresponding control strains. We also tested whether the expression of *IRE1* together with ER folding factors such as *KAR2*, *PDI1*, and *CPR5* would result in synergistic effect, although a small additional increase was overserved for coexpression of *CPR5*, the effect was of a smaller magnitude than one obtained with coexpression of *PSA1* (data not shown). When all four genes were co-expressed final titers decreased again, implying a strong drain on the cellular resource pool. In particular, the co-expression with *IRE1* had strong negative effects on final cell densities (Figures 3, 4), which might result from the additional activation of UPR. Also expressing *IRE1* under control of weaker promotors did not alleviate the costs of *IRE1* coexpression on strain fitness. However despite a reduced fitness, the productivity per amount of cells increased (Supplementary Figure S3). Moreover, a negative correlation between final OD_600_ and increasing plasmid number was observed. Growth of the control strains containing one, two, three, or four plasmids resulted in average final OD_600_ of 6.9, 7.55, 5.74, and 4.89, respectively. In the most severely affected strains expressing the genes, growth reached 90% (one plasmid), 64% (two plasmids), 47% (three plasmids), and 35% (four plasmids) of the final OD_600_ obtained of the corresponding control strains indicating that there is a cumulative effect resulting from the burden of carrying extra plasmids and from expression of the overexpressed proteins.

Interestingly, expression of AP followed a different pattern than expression of antibody. Although we used the same genetic constructs for expression, a single copy of the gene was integrated into the genome at either *LEU2* or *HIS3* locus, expression was controlled by the *GAL1* promoter and the identical signal peptide was used for translocation, the effects of *IRE1*, *PSA1*, *GOT1*, or *HUT1* on the secretion of either the antibody or the AP were very different. As the secreted AP activity, in contrast to the secreted anti-CD20 antibody amounts, increased with increasing induced concentration, we believe that in our set-up the expression of AP is mostly limited at transcriptional level whereas anti-CD20 antibody secretion is limited at the post-translational level (Supplementary Figure S5) and thus expression of *IRE1*, *PSA1*, *GOT1*, or *HUT1* which are all involved in biological processes taking place post-translationally only improve secretion of the antibody.

## Conclusion

In conclusion, by mining transcriptomics data sets from B cell differentiation we identified four new modifications, which enhanced antibody secretion in yeast. Thus, such data exploration is effective in finding novel engineering targets, even when employed to an evolutionary distant organism. So far, comparable experimental approaches had been limited to analyzing the expression host’s own transcriptome and selective overexpression of up-regulated genes (Gasser et al., 2007; Jacobs D.I. et al., 2009). In order to raise the antibody titers to competitive levels further improvements are required. In particular, it will be crucial to identify more engineering targets producing synergistic effects and to use advanced secretion optimized strains as a basis for strain engineering (Wang et al., 2019). Furthermore, it will be essential to generate antibodies with proper N-glycan structures. Unless using post-production *in vitro* glycosylation (Wei et al., 2008; Liu et al., 2018), it needs to be ensured that glycoengineering strategies are compatible with production optimized strains.

## Data Availability Statement

All datasets generated for this study are included in the article/Supplementary Material.

## Author Contributions

AF and EK designed the study and wrote the manuscript. EK conducted the bioinformatics analysis, contributed to cloning of the expression constructs and experimental testing. HI contributed to bioinformatics analysis, cloning of the expression constructs and participated in preliminary experiments. AG created deletion strains and participated in cloning of the expression constructs and preliminary experiments. MP contributed to the gene target selection and cloning of expression constructs. HS contributed to the experimental testing. AF contributed to cloning of the expression constructs, created and analyzed the experimental data. All authors have read the manuscript.

## Conflict of Interest

EK was employed by the company Euretos B.V and HI was employed by Byinfo Oy while the manuscript was finalized. The remaining authors declare that the research was conducted in the absence of any commercial or financial relationships that could be construed as a potential conflict of interest.

## Supplementary Material

FIGURE S1 Selected transcriptomics data sets and comparisons included in the analysis. Gene expression omnibus identifiers for the data sets and microarrays in each sample are shown. Comparisons are shown as arrows and common genes were identified from each of the two phases.

FIGURE S2 Effects of the tested genes on IgG secretion in 96-deep well plate format. Mean antibody titers are displayed for each genetic modification for overexpression strain **(A)** and deletion strains **(B)**. Overexpressed or deleted genes are indicated below the charts. Overexpression and corresponding control strains a derived from YEK66. Strains were grown in SD-Leu medium. Deletion strains are derived from YEK18 and were grown in complete SD medium. Data points of biological replicates together with mean and standard error are shown. *N* = 8, *IRE1*: *N* = 16.

FIGURE S3 Effects of combined expression of *IRE1*, *GOT1*, *HUT1*, and *PSA1* genes on biomass normalized IgG titers. Data from Figure 3 depicting IgG titers normalized by final OD_600_ for strains expressing combinations of two **(A)**, three **(B)**, and four genes **(C)**. Overexpressed genes are indicated below the charts. Data points of biological replicates together with mean and standard error are shown. *N* = 6–8.

FIGURE S4 Optimization of *IRE1* expression. Mean antibody titers **(A,C)** and final cell density **(B,D)** are displayed for three YEK66 derived strains expressing *IRE1* gene under control of P_*TEF1*_, P_*PDI1*_, and P_*KAR2*_ **(A,B)** or *IRE1* gene under control of P_*TEF1*_, P_*PDI1*_, and P_*KAR2*_ together with *GOT1*, *HUT1*, and *PSA1* genes **(C,D)**. Data points of biological replicates together with mean and standard error are shown. *N* = 6.

FIGURE S5 Effect of increasing inducer concentrations on secretion of antibody and AP in 96-deep well plate format. Strain YMH14 was grown in in 96-deep well plate format and expression was induced with 0, 0.5, 1.0, 2.0, and 4.0% galactose. Antibody titers **(A)**, secreted AP activity **(B)** normalized to cell density is reported. Data points of biological replicates together with mean and standard error are shown. *N* = 14–16.

FIGURE S6 Unfolded protein response in strains overexpressing UPR signaling proteins. Growth and activation of UPR under repressive (glucose) and inducing (galactose) conditions. Glucose and galactose were added to the cultures 6 h after inoculation. Results represents mean and standard error of three biological replicates. For each biological replicate four parallel cultures were grown.

TABLE S1 Oligonucleotides used in this study.

TABLE S2 Analysis of the yeast homologs in PC and PB phase and their GO-associations.

TABLE S3 Compilation of numeric data including antibodies titers and final cell densities.

## Abbreviations

LLO: lipid-linked oligosaccharide
PB: plasmablast
PC: plasma cell
UPR: unfolded protein response.

## References

[B1] Agaphonov M. O. Packeiser A. N. Chechenova M. B. Choi E. S. Ter-Avanesyan M. D. (2001). Mutation of the homologue of GDP-mannose pyrophosphorylase alters cell wall structure, protein glycosylation and secretion in *Hansenula polymorpha*. *Yeast* 18 391–402. 10.1002/yea.678 11255248

[B2] Bindea G. Galon J. Mlecnik B. (2013). CluePedia Cytoscape plugin: pathway insights using integrated experimental and in silico data. *Bioinformatics* 29 661–663. 10.1093/bioinformatics/btt019 23325622 PMC3582273

[B3] Bindea G. Mlecnik B. Hackl H. Charoentong P. Tosolini M. Kirilovsky A. (2009). ClueGO: a Cytoscape plug-in to decipher functionally grouped gene ontology and pathway annotation networks. *Bioinformatics* 25 1091–1093. 10.1093/bioinformatics/btp101 19237447 PMC2666812

[B4] Bolstad B. (2004). *Low Level Analysis of High-Density Oligonucleotide Array Data: Background, Normalization And Summarization.* Berkeley, CA: University of California.

[B5] Carlson M. (2016). *org.Sc.sgd.db: Genome Wide Annotation for Yeast.* Avaliable at: http://bioconductor.org/packages/org.Sc.sgd.db. Version: 3.2.3. (accessed July 06, 2017).

[B6] Carlson M. Pages H. (2015). *Hom.Hs.inp.db: Homology Information for Homo Sapiens from Inparanoid, R package version 3.1.2.* (accessed July 06, 2017).

[B7] Cocco M. Stephenson S. Care M. A. Newton D. Barnes N. A. Davison A. (2012). In vitro generation of long-lived human plasma cells. *J. Immunol.* 189 5773–5785. 10.4049/jimmunol.1103720 23162129

[B8] Conchon S. Cao X. Barlowe C. Pelham H. R. B. (1999). Got1p and Sft2p: membrane proteins involved in traffic to the Golgi complex. *EMBO J.* 18 3934–3946. 10.1093/emboj/18.14.3934 10406798 PMC1171469

[B9] Davis S. Meltzer P. S. (2007). GEOquery: a bridge between the gene expression omnibus (GEO) and bioconductor. *Bioinformatics* 14 1846–1847. 10.1093/bioinformatics/btm254 17496320

[B10] de Ruijter J. C. Frey A. D. (2015). Analysis of antibody production in *Saccharomyces cerevisiae*: effects of ER protein quality control disruption. *Appl. Microbiol. Biotechnol.* 99 9061–9071. 10.1007/s00253-015-6807-7 26184977

[B11] de Ruijter J. C. Jurgens G. Frey A. D. (2016a). Screening for novel genes of *Saccharomyces cerevisiae* involved in recombinant antibody production. *FEMS Yeast Res.* 17 1–10. 10.1093/femsyr/fow104 27956492

[B12] de Ruijter J. C. Koskela E. V. Frey A. D. (2016b). Enhancing antibody folding and secretion by tailoring the *Saccharomyces cerevisiae* endoplasmic reticulum. *Microb. Cell Fact.* 15 87. 10.1186/s12934-016-0488-5 27216259 PMC4878073

[B13] de Ruijter J. C. Koskela E. V. Nandania J. Frey A. D. Velagapudi V. (2017). Understanding the metabolic burden of recombinant antibody production in *Saccharomyces cerevisiae* using a quantitative metabolomics approach. *Yeast* 35 1–11. 10.1002/yea.3298 29159981

[B14] Dinnis D. M. James D. C. (2005). Engineering mammalian cell factories for improved recombinant monoclonal antibody production: lessons from nature? *Biotechnol. Bioeng.* 91 180–189. 10.1002/bit.20499 15880827

[B15] Dunning M. Lynch A. Eldridge M. (2015). *Illuminahumanv4.db: Illumina HumanHT12v4 Annotation data (chip illuminaHumanv4). R Package Version 1.26.0*.

[B16] Gasser B. Sauer M. Maurer M. Stadlmayr G. Mattanovich D. (2007). Transcriptomics-based identification of novel factors enhancing heterologous protein secretion in yeasts. *Appl. Env. Microbiol.* 73 6499–6507. 10.1128/aem.01196-07 17766460 PMC2075068

[B17] Gietz D. St Jean A. Woods R. A. Schiestl R. H. (1992). Improved method for high efficiency transformation of intact yeast cells. *Nucleic Acid Res.* 20:1425. 10.1093/nar/20.6.1425 1561104 PMC312198

[B18] Güldener U. Heck S. Fiedler T. Beinhauer J. Hegemann J. H. (1996). A new efficient gene disruption cassette for repeated use in budding yeast. *Nucleic Acids Res.* 24 2519–2524. 10.1093/nar/24.13.2519 8692690 PMC145975

[B19] Hackel B. J. Huang D. Bubolz J. C. Wang X. X. Shusta E. V. (2006). Production of soluble and active transferrin receptor-targeting single-chain antibody using *Saccharomyces cerevisiae*. *Pharm. Res.* 23 790–797. 10.1007/s11095-006-9778-7 16550469

[B20] Hendershot L. M. Sitia R. (2004). “Immunoglobulin assembly and secretion,” in *Molecular Biology of B Cells*, eds Reth M. Radbruch A. Alt F. Honjo T. Neuberger M. (Amsterdam: Elsevier), 261–273. 10.1016/b978-012053641-2/50018-6

[B21] Idiris A. Tohda H. Kumagai H. Takegawa K. (2010). Engineering of protein secretion in yeast: strategies and impact on protein production. *Appl. Microbiol. Biotechnol.* 86 403–417. 10.1007/s00253-010-2447-0 20140428

[B22] Jacobs D. I. Olsthoorn M. M. Maillet I. Akeroyd M. Breestraat S. Donkers S. (2009). Effective lead selection for improved protein production in *Aspergillus niger* based on integrated genomics. *Fungal. Genet. Biol.* 46 (Suppl. 1), S141–S152. 10.1016/j.fgb.2008.08.012 18824119

[B23] Jacobs P. P. Geysens S. Vervecken W. Contreras R. Callewaert N. (2009). Engineering complex-type N-glycosylation in *Pichia pastoris* using GlycoSwitch technology. *Nat. Protoc.* 4 58–70. 10.1038/nprot.2008.213 19131957

[B24] Janik A. Sosnowska M. Kruszewska J. Krotkiewski H. Lehle L. (2003). Overexpression of GDP-mannose pyrophosphorylase in *Saccharomyces cerevisiae* corrects defects in dolichol-linked saccharide formation and protein glycosylation. *Biochim. Biophys. Acta* 1621 22–30. 10.1016/s0304-4165(03)00026-6 12667607

[B25] Kainuma M. Chiba Y. Takeuchi M. Jigami Y. (2001). Overexpression of HUT1 gene stimulates in vivo galactosylation by enhancing UDP – galactose transport activity in *Saccharomyces cerevisiae*. *Yeast* 18 533–541. 10.1002/yea.708 11284009

[B26] Kimata Y. Ishiwata-Kimata Y. Ito T. Hirata A. Suzuki T. Oikawa D. (2007). Two regulatory steps of ER-stress sensor Ire1 involving its cluster formation and interaction with unfolded proteins. *J. Cell Biol.* 179 75–86. 10.1083/jcb.200704166 17923530 PMC2064738

[B27] Koskela E. V. de Ruijter J. C. Frey A. D. (2017). Following nature’s roadmap: folding factors from plasma cells led to improvements in antibody secretion in *S. cerevisiae*. *Biotechnol. J.* 12 1–13. 10.1002/biot.201600631 28429845

[B28] Koskela E. V. Frey A. D. (2015). Homologous recombinatorial cloning without the creation of single-stranded ends: exonuclease and ligation-independent cloning (ELIC). *Mol. Biotechnol.* 57 233–240. 10.1007/s12033-014-9817-2 25370826

[B29] Laukens B. De Visscher C. Callewaert N. (2015). Engineering yeast for producing human glycoproteins: where are we now? *Future Microbiol.* 10 21–34. 10.2217/fmb.14.104 25598335 PMC7617146

[B30] Le Gallou S. Caron G. Delaloy C. Rossille D. Tarte K. Fest T. (2012). IL-2 requirement for human plasma cell generation: coupling differentiation and proliferation by enhancing MAPK–ERK signaling. *J. Immunol.* 189 161–173. 10.4049/jimmunol.1200301 22634617

[B31] Li H. Sethuraman N. Stadheim T. A. Zha D. Prinz B. Ballew N. (2006). Optimization of humanized IgGs in glycoengineered *Pichia pastoris*. *Nat. Biotechnol.* 24 210–215. 10.1038/nbt1178 16429149

[B32] Liu C. P. Tsai T. I. Cheng T. Shivatare V. S. Wu C. Y. Wu C. Y. (2018). Glycoengineering of antibody (Herceptin) through yeast expression and in vitro enzymatic glycosylation. *Proc. Natl. Acad. Sci. U.S.A.* 115 720–725. 10.1073/pnas.1718172115 29311294 PMC5789950

[B33] Lorente-Rodríguez A. Heidtman M. Barlowe C. (2009). Multicopy suppressor analysis of thermosensitive YIP1 alleles implicates GOT1 in transport from the ER. *J. Cell Sci.* 122 1540–1550. 10.1242/jcs.042457 19383723 PMC2680100

[B34] Love K. R. Politano T. J. Panagiotou V. Jiang B. Stadheim T. A. Love J. C. (2012). Systematic single-cell analysis of *Pichia pastoris* reveals secretory capacity limits productivity. *PLoS One* 7:e37915. 10.1371/journal.pone.0037915 22685548 PMC3369916

[B35] Malsam J. Kreye S. Söllner T. H. (2008). Membrane traffic in the secretory pathway: Membrane fusion: SNAREs and regulation. *Cell. Mol. Life Sci.* 65 2814–2832. 10.1007/s00018-008-8352-3 18726177 PMC11131752

[B36] Mumberg D. Muller R. Funk M. (1994). Regulatable promoters of *Saccharomyces cerevisiae*: comparison of transcriptional activity and their use for heterologous expression. *Nucleic Acids Res.* 22 5767–5768. 10.1093/nar/22.25.5767 7838736 PMC310147

[B37] Mumberg D. Müller R. Funk M. (1995). Yeast vectors for the controlled expression of heterologous proteins in different genetic backgrounds. *Gene* 156 119–122. 10.1016/0378-1119(95)00037-7 7737504

[B38] Nakanishi H. Nakayama K. I. Yokota A. Tachikawa H. Takahashi N. Jigami Y. (2001). HutI proteins identified in *Saccharomyces cerevisiae* and *Schizosaccharomyces pombe* are functional homologues involved in the protein-folding process at the endoplasmic reticulum. *Yeast* 18 543–554. 10.1002/yea.707 11284010

[B39] Nasab F. P. Aebi M. Bernhard G. Frey A. D. (2013). A combined system for engineering glycosylation efficiency and glycan structure in *Saccharomyces cerevisiae*. *Appl. Environ. Microbiol.* 79 997–1007. 10.1128/AEM.02817-12 23204425 PMC3568548

[B40] Oracki S. A. Walker J. A. Hibbs M. L. Corcoran L. M. Tarlinton D. M. Tarlinton D. M. (2010). Plasma cell development and survival. *Immunol. Rev.* 237 140–159. 10.1111/j.1600-065x.2010.00940.x 20727034

[B41] Pages H. Carlson M. Falcon S. Li N. (2016). *AnnotationDbi: Annotation Database Interface. R Package Version 1.34.4.*

[B42] Payne T. Finnis C. Evans L. R. Mead D. J. Avery S. V. Archer D. B. (2008). Modulation of chaperone gene expression in mutagenized *Saccharomyces cerevisiae* strains developed for recombinant human albumin production results in increased production of multiple heterologous proteins. *Appl Env. Microbiol.* 74 7759–7766. 10.1128/AEM.01178-08 18931293 PMC2607181

[B43] Piirainen M. A. Boer H. De Ruijter J. C. Frey A. D. (2016). A dual approach for improving homogeneity of a human-type N-glycan structure in *Saccharomyces cerevisiae*. *Glycoconj. J.* 33 189–199. 10.1007/s10719-016-9656-4 26983412

[B44] Pincus D. Chevalier M. W. Aragón T. van Anken E. Vidal S. E. El-Samad H. (2010). BiP binding to the ER-stress sensor Ire1 tunes the homeostatic behavior of the unfolded protein response. *PLoS Biol.* 8:e1000415. 10.1371/journal.pbio 20625545 PMC2897766

[B45] Powers D. B. Amersdorfer P. Poul M.-A. Nielsen U. B. Shalaby M. R. Adams G. P. (2001). Expression of single-chain Fv-Fc fusions in *Pichia pastoris*. *J. Immunol. Methods* 251 123–135. 10.1016/s0022-1759(00)00290-8 11292488

[B46] Rakestraw J. A. Sazinsky S. L. Piatesi A. Antipov E. Wittrup K. D. (2009). Directed evolution of a secretory leader for the improved expression of heterologous proteins and full-length antibodies in *Saccharomyces cerevisiae*. *Biotechnol. Bioeng.* 103 1192–1201. 10.1002/bit.22338 19459139 PMC2847895

[B47] Romijn E. P. Christis C. Wieffer M. Gouw J. W. Fullaondo A. van der Sluijs P. (2005). Expression clustering reveals detailed co-expression patterns of functionally related proteins during B cell differentiation. *Mol. Cell. Proteomics* 4 1297–1310. 10.1074/mcp.m500123-mcp200 15961381

[B48] Sanchez-Garcia L. Martín L. Mangues R. Ferrer-Miralles N. Vázquez E. Villaverde A. (2016). Recombinant pharmaceuticals from microbial cells: a 2015 update. *Microb. Cell Fact.* 15 33. 10.1186/s12934-016-0437-3 26861699 PMC4748523

[B49] Shaffer A. L. Shapiro-Shelef M. Iwakoshi N. N. Lee A. H. Qian S. B. Zhao H. (2004). XBP1, downstream of Blimp-1, expands the secretory apparatus and other organelles, and increases protein synthesis in plasma cell differentiation. *Immunity* 21 81–93. 10.1016/j.immuni.2004.06.010 15345222

[B50] Shannon P. Markiel A. Ozier O. Baliga N. Wang J. Ramage D. (2003). Cytoscape: a software environment for integrated models of biomolecular interaction networks. *Genome Res.* 13 2498–2504. 10.1101/gr.1239303 14597658 PMC403769

[B51] Sheng J. Flick H. Feng X. (2017). Systematic optimization of protein secretory pathways in *Saccharomyces cerevisiae* to increase expression of hepatitis B Small Antigen. *Front. Microbiol.* 8:875. 10.3389/fmicb.2017.00875 28559891 PMC5432677

[B52] Shusta E. V. Raines R. T. Pluckthun A. Wittrup K. D. (1998). Increasing the secretory capacity of *Saccharomyces cerevisiae* for production of single-chain antibody fragments. *Nat Biotechnol* 16 773–777. 10.1038/nbt0898-773 9702778

[B53] Sidrauski C. Walter P. (1997). The transmembrane kinase Ire1p is a site-specific endonuclease that initiates mRNA splicing in the unfolded protein response. *Cell* 90 1031–1039. 10.1016/s0092-8674(00)80369-4 9323131

[B54] Smith-Gill S. J. Mainhart C. R. Lavoie T. B. Rudikoff S. Potter M. (1984). VL-VH expression by monoclonal antibodies recognizing avian lysozyme. *J. Immunol.* 132 963–967. 6418816

[B55] Tarlinton D. Radbruch A. Hiepe F. Dörner T. (2008). Plasma cell differentiation and survival. *Curr. Opin. Immunol.* 20 162–169. 10.1016/j.coi.2008.03.016 18456483

[B56] Taxis C. Knop M. (2006). System of centromeric, episomal, and integrative vectors based on drug resistance markers for *Saccharomyces cerevisiae*. *Biotechniques* 40 73–78. 10.2144/000112040 16454043

[B57] Uccelletti D. Staneva D. Rufini S. Venkov P. Palleschi C. (2005). Enhanced secretion of heterologous proteins in *Kluyveromyces lactis* by overexpression of the GDP-mannose pyrophosphorylase. *KlPsa1p. FEMS Yeast Res.* 5 735–746. 10.1016/j.femsyr.2005.01.004 15851102

[B58] Valkonen M. Penttilä M. Saloheimo M. (2003a). Effects of inactivation and constitutive expression of the unfolded- protein response pathway on protein production in the yeast *Saccharomyces cerevisiae*. *Appl. Environ. Microbiol.* 69 2065–2072. 10.1128/aem.69.4.2065-2072.2003 12676684 PMC154816

[B59] Valkonen M. Ward M. Wang H. Penttilä M. Saloheimo M. (2003b). Improvement of foreign-protein production in *Aspergillus niger* var. awamori by Constitutive Induction of the Unfolded-Protein Response. *Appl. Environ. Microbiol.* 69 6979–6986. 10.1128/aem.69.12.6979-6986.2003 14660339 PMC309985

[B60] Van Anken E. Romijn E. P. Maggioni C. Mezghrani A. Sitia R. Braakman I. (2003). Sequential waves of functionally related proteins are expressed when B cells prepare for antibody secretion. *Immunity* 18 243–253. 10.1016/s1074-7613(03)00024-4 12594951

[B61] Victora G. D. Nussenzweig M. C. (2012). Germinal centers. *Annu. Rev. Immunol.* 30 429–457. 10.1146/annurev-immunol-020711-075032 22224772

[B62] Walsh G. (2014). Biopharmaceutical benchmarks 2014. *Nat. Biotechnol.* 32 992–1000. 10.1038/nbt.3040 25299917

[B63] Wang G. Björk S. M. Huang M. Liu Q. Campbell K. Nielsen J. (2019). RNAi expression tuning, microfluidic screening, and genome recombineering for improved protein production in *Saccharomyces cerevisiae*. *Proc. Natl. Acad. Sci. U.S.A.* 116 9324–9332. 10.1073/pnas.1820561116 31000602 PMC6511059

[B64] Wei Y. Li C. Huang W. Li B. Strome S. Wang L. X. (2008). Glycoengineering of human IgG1-Fc through combined yeast expression and in vitro chemoenzymatic glycosylation. *Biochemistry* 47 10294–10304. 10.1021/bi800874y 18771295 PMC2628294

[B65] Zhang B. Chang A. Kjeldsen T. B. Arvan P. (2001). Intracellular retention of newly synthesized insulin in yeast is caused by endoproteolytic processing in the Golgi complex. *J. Cell Biol.* 153 1187–1198. 10.1083/jcb.153.6.1187 11402063 PMC2192022

