# Supplementary figures and images for: Mining Data From Plasma Cell Differentiation Identified Novel Genes for Engineering of a Yeast Antibody Factory
Source: Front Bioeng Biotechnol. 2020 Mar 31;8:255. doi: 10.3389/fbioe.2020.00255 (PMC7136540; doi:10.3389/fbioe.2020.00255)

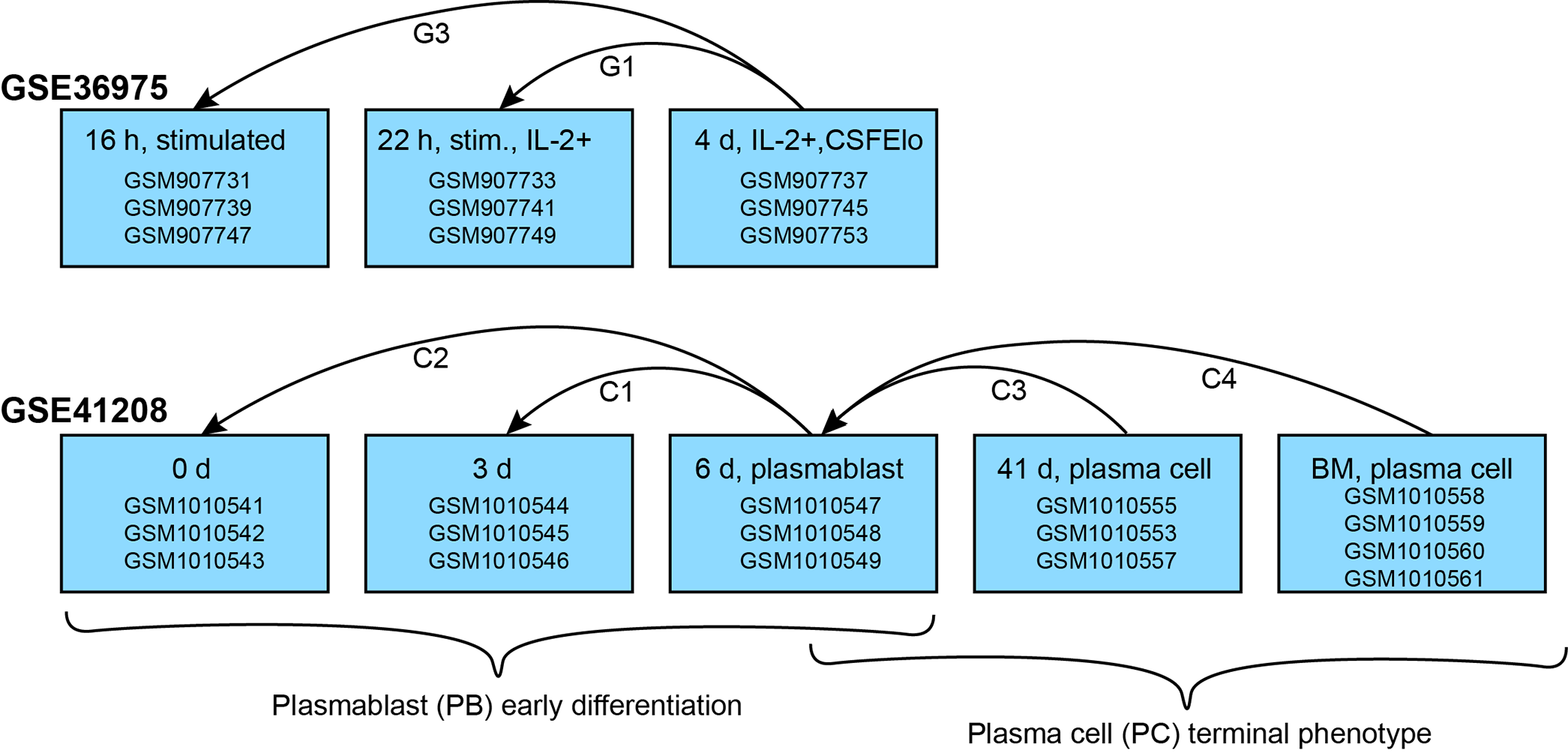

Supplement: FIGURE S1 — Selected transcriptomics data sets and comparisons included in the analysis. Gene expression omnibus identifiers for the data sets and microarrays in each sample are shown. Comparisons are shown as arrows and common genes were identified from each of the two phases. [file Image_1.TIF]
