# Supplementary material for: Mining Data From Plasma Cell Differentiation Identified Novel Genes for Engineering of a Yeast Antibody Factory
Source: Front Bioeng Biotechnol. 2020 Mar 31;8:255. doi: 10.3389/fbioe.2020.00255 (PMC7136540; doi:10.3389/fbioe.2020.00255)
Supplement: TABLE S1 — Oligonucleotides used in this study. [file Table_1.DOCX]

**Supplementary Materials, S1: Oligonucleotides used in this study.**

| **Name** | **Sequence (5' to 3')** | **Target** |
| --- | --- | --- |
| 1 | GATAGTCTCAAGATACAACTGAGAAGAAGCACAATTATGTCCGATAGATTGTACGCTGCAGGTCGACAAC | 5' for *ATE1* knock-out, loxP site from pUG6 |
| 2 | TTAGGATTGTTATTAGAATAGTGTGTTGAAGGCTCACATTTGCTCACTATGGCCACTAGTG GATCTGATATC | 3' for *ATE1* knock-out, loxP site from pUG6 |
| 3 | GATTAGTGATGGATTTCTAAGTGGCAGCGTTGAAAGATCGTGCAAAGGTTGTACGCTGCAGGTCGACAAC | 5' for *DPH2* knock-out, loxP site from pUG6 |
| 4 | AATAGAGAAGTCGAGGGAAACAAATTATAAGAGTCATTTGTTTTCCTTTGGCCACTAGTGGATCTGATATC | 3' for *DPH2* knock-out, loxP site from pUG6 |
| 5 | GGATGAATATGGAATGGTCATTGGGCAAAAGGTCCTGCTAGCTCGTATACGTACGCTGCAGGTCGACAAC | 5' for *UTR4* knock-out, loxP site from pUG6 |
| 6 | GATTATACTGCTTTTAGCGCATATATACAGTTTTGTTAAAGAGTTTCAAAGGCCACTAGTGGATCTGATATC | 3' for *UTR4* knock-out, loxP site from pUG6 |
| 7 | AACTAGTGAGACC ATG TGGCTCACAGAGGCTC | 5' for *GOT1*, to create overexpression plasmid, common ends |
| 8 | CTAATTACATGACTCGAG TTATACTGGCAGAACCCTAACTC | 3' for *GOT1*, to create overexpression plasmid, common ends |
| 9 | AACTAGTGAGACC ATG GCACACTATCCACCTTC | 5' for *GSH2*, to create overexpression plasmid, common ends |
| 10 | CTAATTACATGACTCGAGCTAGTAAAGAATAATACTGTCCAAACATC | 3' for *GSH2*, to create overexpression plasmid, common ends |
| 11 | AACTAGTGAGACC ATGAAAGGTTTAATTTTAGTCGGTGGTTAC | 5' for *PSA1*, to create overexpression plasmid, common ends |
| 12 | CTAATTACATGACTCGAGTCACATAATAATAGCTTCCTTTGGAAC | 3' for *PSA1*, to create overexpression plasmid, common ends |
| 13 | AACTAGTGAGACC ATGACTGACACAAAACAGC | 5' for *QRI1*, to create overexpression plasmid, common ends |
| 14 | CTAATTACATGACTCGAGTTATTTTTCTAATACTATACCACTTCTGTC | 3' for *QRI1*, to create overexpression plasmid, common ends |
| 15 | AACTAGTGAGACC ATGAGAGCGTTGAGATTCC | 5' for *ALG5*, to create overexpression plasmid, common ends |
| 16 | CTAATTACATGACTCGAGCTAACATTTCTTATTATCTCTATATATCCC | 3' for *ALG5*, to create overexpression plasmid, common ends |
| 17 | AACTAGTGAGACC ATGGCGGGAAGTACATCCAG | 5' for *HUT1*, to create overexpression plasmid, common ends |
| 18 | CTAATTACATGACTCGAGCTACGCAGATTTTGCCTTCG | 3' for *HUT1*, to create overexpression plasmid, common ends |
| 19 | AACTAGTGAGACC ATGTTGCGACTTTTTTCACTGG | 5' for *ALG7*, to create overexpression plasmid, common ends |
| 20 | CTAATTACATGACTCGAGTCAACGTACTGTCCATAGGTTG | 3' for *ALG7*, to create overexpression plasmid, common ends |
| 21 | GCAATCTAATCTAAGTTTTCTAGAACTAGTGAGACCATG | Complementary to the TEF-promoter (5' end) and common RE-sites (3' end) |
| 22 | GCGTGACATAACTAATTACATGACTCGAG | Complementary to CYC1-terminator (5' end) |
| OAF33 | AAACTAGTATGTCTCATCACAAGAAACGTG | 5' for *SEC24*, to create overexpression plasmid, with SpeI-site |
| OAF34 | AACTCGAGTTATTTGCTAATTCTGGCTTTCAT | 3' for *SEC24*, to create overexpression plasmid, with XhoI-site |
| OAF35 | AAACTAGTATGCGTCTACTTCGAAGAAACA | 5' for *IRE1*, to create overexpression plasmid, with SpeI-site |
| OAF36 | AACTCGAGTTATGAATACAAAAATTCACGTAAAAT | 3' for *IRE1*, to create overexpression plasmid, with XhoI-site |
| OAF37 | AAACTAGTATGGATGCTGTAATACTGAATCT | 5' for *DER1*, to create overexpression plasmid, with SpeI-site |
| OAF38 | AACTCGAGTTAGGGTGTTTCAGTGTTGCG | 3' for *DER1*, to create overexpression plasmid, with XhoI-site |
| OAF41 | AAACTAGTATGTCAGGTCCTCCACCTCC | 5' for *Bxi1*, to create overexpression plasmid, with SpeI-site |
| OAF42 | AACTCGAGTTAATTGTCGTCGTTAGAGTTG | 3' for *Bxi1*, to create overexpression plasmid, with XhoI-site |
| OAF219 | AAACTAGTATGTTTAAATCTGTTGTTTATTC | 5' for *Pho5*, to create overexpression plasmid, with SpeI-site |
| OAF220 | AACTCGAGTTAATGGTGATGGTGATGGTGTTGTCTCAATAGACTGGCG | 3' for *Pho5*, to create overexpression plasmid, with XhoI-site |
| OJR38 | ATGTTCAGCTTGAAAGCATTATTGCCATTGGCCTTGTTGTTGGTCAGCGCGAGGCCCAGAATACCCTCC | *PEP4* deletion |
| OJR39 | TCAAATTGCTTTGGCCAAACCAACCGCATTGTTGCCCAAATCGTAAATAGCACTGGATGGCGGCGTTAG | *PEP4* deletion |
| OJR40 | AATGCGAATACGGGGAAC | confirmation of *PEP4* deletion |
| EK005 | ATGAAGTTAGAAAATACTCTATTTACACTCGGTGCCCTAGGGAGCATCTCGTACGCTGCAGGTCGACAAC | *PRB1* deletion |
| EK006 | TTAAATAATATTCAATTTATCAAGAATATCTCTCACTTGATCAAAGATTACCACTAGTGGATCTGATATC | *PRB1* deletion |
| EK158 | CTCAGTAATGCCACTGCAG | confirmation of *PRB1* deletion |
